# Data on the key performance indicators for quality of service of GSM networks in Nigeria

**DOI:** 10.1016/j.dib.2017.12.005

**Published:** 2017-12-14

**Authors:** Segun I. Popoola, Aderemi A. Atayero, Nasir Faruk, Joke A. Badejo

**Affiliations:** a Department of Electrical and Information Engineering, Covenant University, Ota, Nigeria; b Department of Telecommunication Science, University of Ilorin, Ilorin, Nigeria

**Keywords:** Quality of service, GSM networks, Call setup success rate, Drop call rate, Stand-alone dedicated channel congestion, Traffic channel congestion

## Abstract

In this data article, the Key Performance Indicators (KPIs) for Quality of Service (QoS) of Global System for Mobile Communications (GSM) networks in Nigeria are provided and analyzed. The data provided in this paper contain the Call Setup Success Rate (CSSR), Drop Call Rate (DCR), Stand-alone Dedicated Channel (SDCCH) congestion, and Traffic Channel (TCH) congestion for the four GSM network operators in Nigeria (Airtel, Etisalat, Glo, and MTN). These comprehensive data were obtained from the Nigerian Communications Commission (NCC). Significant differences in each of the KPIs for the four quarters of each year were presented based on Analysis of Variance (ANOVA). The values of the KPIs were plotted against the months of the year for better visualization and understanding of data trends across the four quarters. Multiple comparisons of the mean-quarterly differences of the KPIs were also presented using Tukey's Post Hoc test. Public availability and further interpretation and discussion of these useful information will assist the network providers, Nigerian government, local and international regulatory bodies, policy makers, and other stakeholders in ensuring access of people, machines, and things to high quality telecommunications services.

**Specifications Table**

**Table:** 

Subject area	*Telecommunication Engineering*
More specific subject area	*Cellular/Mobile Networks*
Type of data	*Table and figure*
How data was acquired	*Unprocessed secondary data*
Data format	*Filtered and analyzed*
Experimental factors	*Data were obtained from Nigerian Communications Commission (NCC)*
Experimental features	*The KPIs were measured from the Network Operating Centres (NOCs) of Airtel, Etisalat, Glo, and MTN at busy hours at the Base Station Controller (BSC) layer of the GSM networks. Computational analysis of the data are further provided.*
Data source location	*The data covers all the GSM networks deployed by the operators across Nigeria*
Data accessibility	*Data are available within this article*
Software	*MATLAB 2016a*

**Value of the data**

• The mobile network providers, the Nigerian government, local and international regulatory bodies, telecommunication policy makers, and other stakeholders in the telecommunication industry in Nigeria, Africa, and the world will find the analyses of the data provided in this article to be most useful [1].
• The importance of the analysis of these data is usually needful for appropriate regulations and quality assurance [2].
• Researchers in both academia and telecommunication industry can further explore and interpret the data provided in this article to solve QoS-related issues in GSM networks [3], [4], [5], [6], [7], [8], [9], [10], [11], [12].
• The major trends in these data and the statistical analyses will help GSM network subscribers to benchmark the services offered by the mobile network operators [13], [14], [15].
• Contextual interpretation and discussion of the data will help mobile network operators to gain accurate and deep understanding of the QoS offered across the months and quarters of the year [16].

## 1 Data

Accurate radio network planning is essential for good Quality of Service (QoS) [16], [17], [18]. The Key Performance Indicators (KPIs) for QoS of Global System for Mobile Communications (GSM) networks in Nigeria presented in this article were collected from Nigerian Communications Commission (NCC). These KPIs include Call Setup Success Rate (CSSR), Drop Call Rate (DCR), Stand-alone Dedicated Channel (SDCCH) congestion, and Traffic Channel (TCH) congestion for the four GSM network operators in Nigeria (Airtel, Etisalat, Glo, and MTN). The raw data were measured during busy hours at the Base Station Controller (BSC) layer and analyzed based on monthly and quarterly mean values to gain useful insights on the QoS provided by each of the mobile network operators. The data covers KPIs that were measured monthly from January, 2014 to December, 2016.

**Fig. 1:**
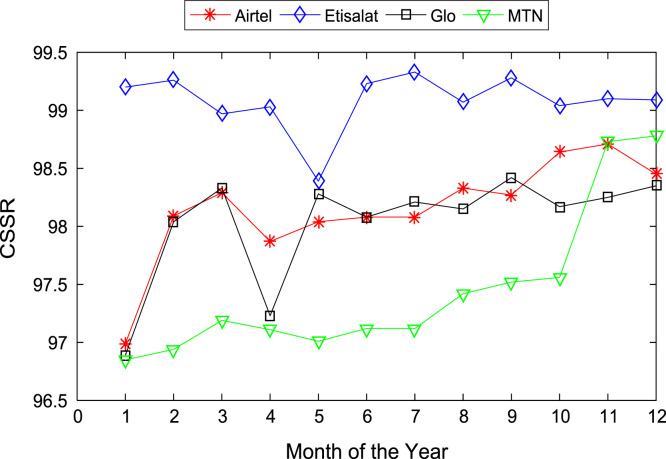
Monthly mean CSSR for the mobile network operators in 2014.

**Fig. 2:**
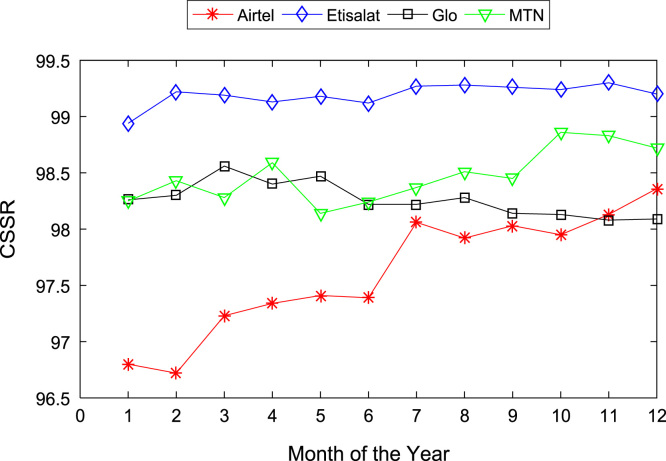
Monthly mean CSSR for the mobile network operators in 2015.

**Fig. 3:**
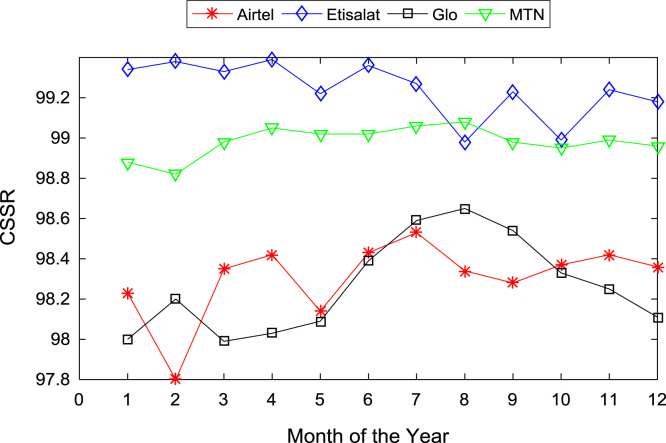
Monthly mean CSSR for the mobile network operators in 2016.

**Fig. 4:**
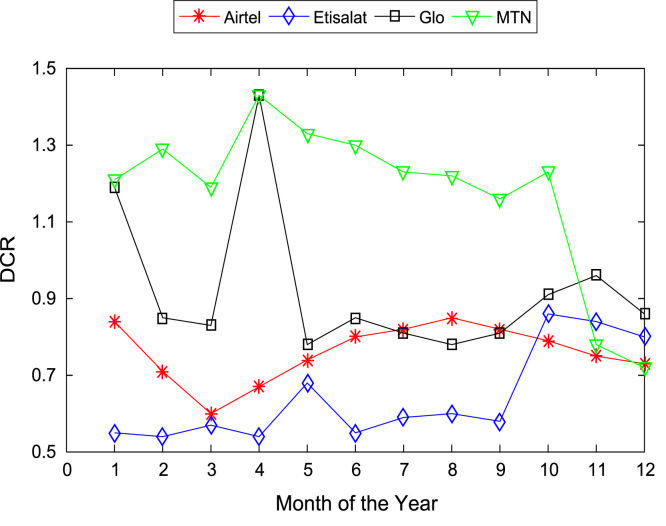
Monthly mean DCR for the mobile network operators in 2014.

**Fig. 5:**
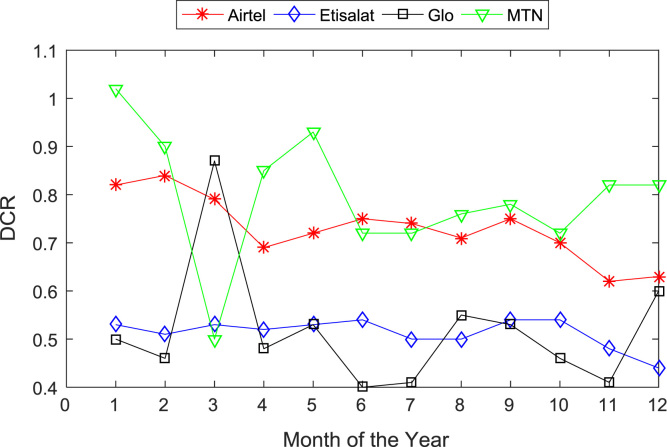
Monthly mean DCR for the mobile network operators in 2015.

**Fig. 6:**
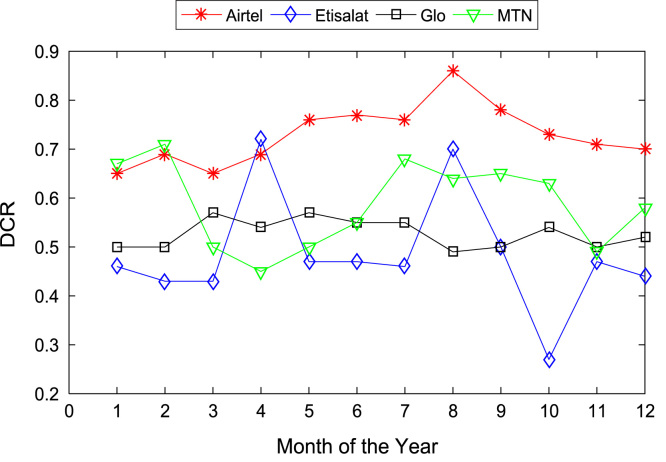
Monthly mean DCR for the mobile network operators in 2016.

**Fig. 7:**
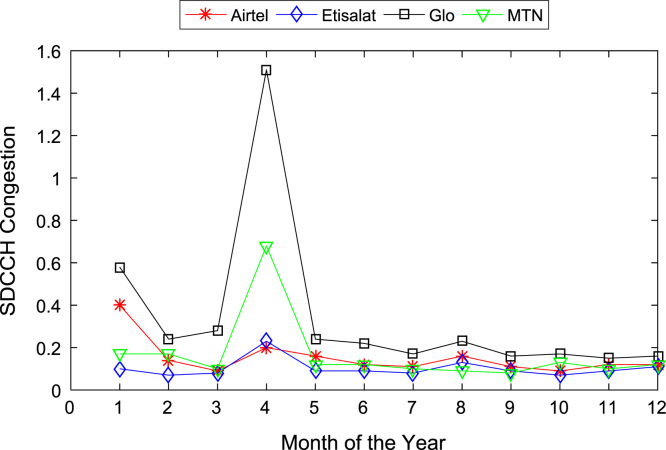
Monthly mean SDCCH congestion for the mobile network operators in 2014.

**Fig. 8:**
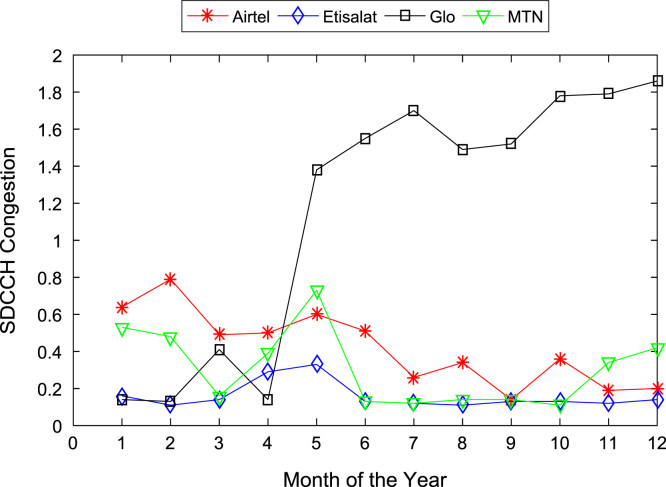
Monthly mean SDCCH congestion for the mobile network operators in 2015.

**Fig. 9:**
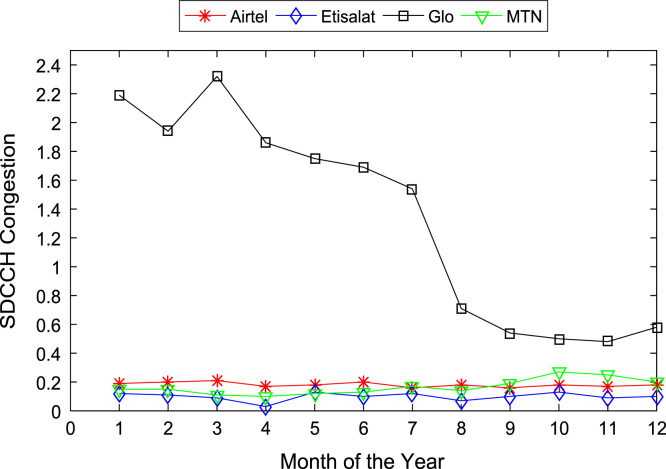
Monthly mean SDCCH congestion for the mobile network operators in 2016.

**Fig. 10:**
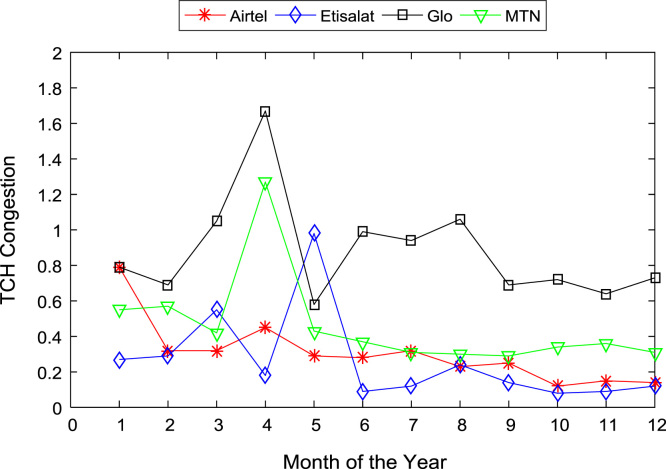
Monthly mean TCH congestion for the mobile network operators in 2014.

**Fig. 11:**
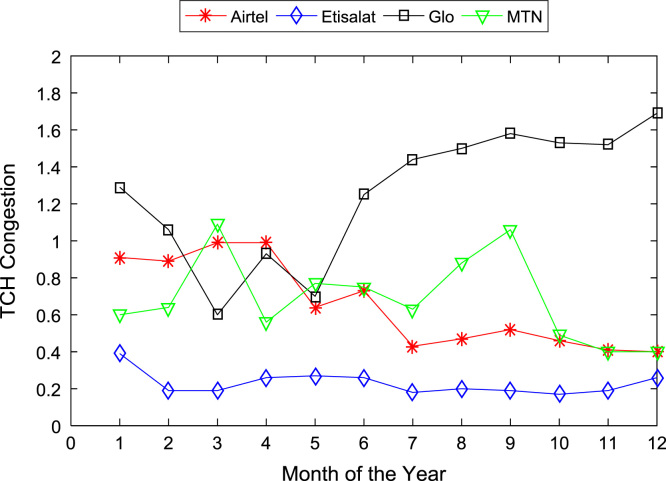
Monthly mean TCH congestion for the mobile network operators in 2015.

**Fig. 12:**
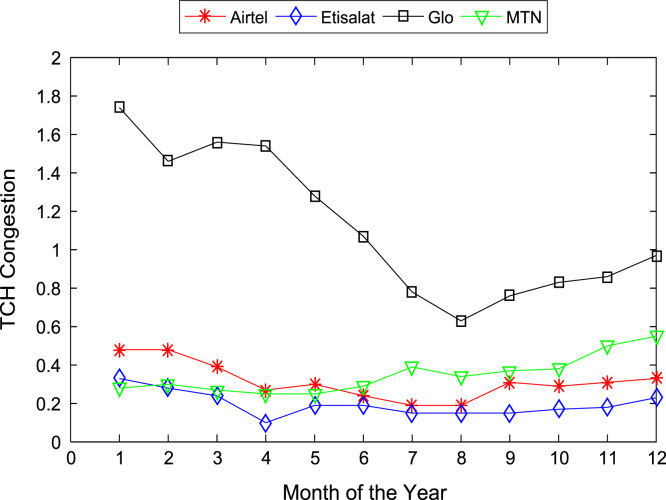
Monthly mean TCH congestion for the mobile network operators in 2016.

Table 1, Table 2 present the summary of the general descriptive statistics (total number of samples, mean, median. mode, minimum, maximum, mean absolute deviation, standard deviation, first and third quartile, kurtosis, and skewness) of the dataset. In addition, Fig. 1, Fig. 2, Fig. 3, Fig. 4, Fig. 5, Fig. 6, Fig. 7, Fig. 8, Fig. 9, Fig. 10, Fig. 11, Fig. 12 show the trends of monthly variations in CSSR, DCR, SDCCH congestion, and TCH congestion for Airtel, Etisalat, Glo, and MTN throughout the three-year data coverage.

**Table 1:** Measure of central tendency of QoS KPIs of GSM network operators.

**QoS Index**	**Mobile network operator**	**Total sample**	**Mean**	**Median**	**Mode**	**Min**	**Max**
**CSSR**	Airtel	36	98.024	98.135	98.08	96.720	98.710
	Etisalat	36	99.173	99.22	99.18	98.390	99.390
	Glo	36	98.187	98.22	98.08	96.890	98.650
	MTN	36	98.300	98.55	97.12	96.850	99.080
**DCR**	Airtel	36	0.740	0.740	0.690	0.600	0.860
	Etisalat	36	0.547	0.530	0.540	0.270	0.860
	Glo	36	0.655	0.550	0.500	0.400	1.430
	MTN	36	0.852	0.770	0.720	0.450	1.430
**SDCCH congestion**	Airtel	36	0.251	0.180	0.160	0.090	0.790
	Etisalat	36	0.120	0.110	0.090	0.030	0.330
	Glo	36	0.947	0.580	0.140	0.130	2.320
	MTN	36	0.213	0.140	0.120	0.080	0.730
**TCH congestion**	Airtel	36	0.424	0.325	0.320	0.120	0.990
	Etisalat	36	0.229	0.190	0.190	0.080	0.980
	Glo	36	1.087	1.020	0.690	0.580	1.740
	MTN	36	0.499	0.400	0.250	0.250	1.270

**Table 2:** Measure of data dispersion of QoS KPIs of GSM network operators.

	**Mobile network operator**	**Mean absolute deviation**	**Standard deviation**	**Q1**	**Q3**	**Kurtosis**	**Skewness**
**CSSR**	Airtel	0.380	0.505	97.895	98.360	3.536	−1.180
	Etisalat	0.124	0.181	99.095	99.275	10.831	−2.337
	Glo	0.199	0.327	98.090	98.340	9.717	−2.276
	MTN	0.636	0.756	97.540	98.955	2.009	−0.741
**DCR**	Airtel	0.054	0.067	0.695	0.790	2.310	−0.085
	Etisalat	0.082	0.119	0.470	0.575	4.285	0.911
	Glo	0.190	0.232	0.500	0.820	4.913	1.411
	MTN	0.242	0.287	0.645	1.175	1.951	0.497
**SDCCH congestion**	Airtel	0.132	0.173	0.150	0.300	4.570	1.578
	Etisalat	0.035	0.057	0.090	0.130	8.291	2.184
	Glo	0.706	0.753	0.225	1.695	1.427	0.290
	MTN	0.121	0.165	0.120	0.225	5.534	1.861
**TCH congestion**	Airtel	0.183	0.240	0.275	0.480	3.281	1.111
	Etisalat	0.091	0.157	0.150	0.260	15.805	3.298
	Glo	0.324	0.372	0.745	1.480	1.651	0.303
	MTN	0.191	0.252	0.310	0.585	4.595	1.481

## 2 Materials and methods

The relationships between CSSR, DCR, SDCCH congestion, and TCH congestion of Airtel, Etisalat, Glo, and MTN were estimated using linear correlation. The correlation matrices are presented in Table 3, Table 4, Table 5, Table 6. ANOVA tests were also performed for all the QoS KPIs presented in this data article to identify the differences among the quarterly-means for each of the mobile network operators. Table 7, Table 8, Table 9, Table 10 presents the ANOVA test results for CSSR, DCR, SDCCH congestion, and TCH congestion respectively. The significant differences in the quarterly-means of the QoS KPIs were further investigated based on multiple comparison using Tukey's Post Hoc test at 95% Confidence Interval. The results of the comparisons are presented in Table 11, Table 12, Table 13. The data analyzed in this article are made available in Table 14, Table 15, Table 16, Table 17, Table 18.

**Table 3:** Correlation matrix for CSSR.

**Mobile network operator**	**Airtel**	**Etisalat**	**Glo**	**MTN**
**Airtel**	1			
**Etisalat**	0.071152119	1		
**Glo**	0.195841509	−0.067886319	1	
**MTN**	0.234379201	0.362204336	0.418815939	1

**Table 4:** Correlation matrix for DCR.

**Mobile network operator**	**Airtel**	**Etisalat**	**Glo**	**MTN**
**Airtel**	1			
**Etisalat**	0.279793691	1		
**Glo**	0.144183419	0.409243609	1	
**MTN**	0.199628489	0.29964156	0.651951552	1

**Table 5:** Correlation matrix for SDCCH congestion.

**Mobile network operator**	**Airtel**	**Etisalat**	**Glo**	**MTN**
**Airtel**	1			
**Etisalat**	0.524717639	1		
**Glo**	−0.036816239	0.093673675	1	
**MTN**	0.565437362	0.752745819	0.025714345	1

**Table 6:** Correlation matrix for TCH congestion.

**Mobile network operator**	**Airtel**	**Etisalat**	**Glo**	**MTN**
**Airtel**	1			
**Etisalat**	0.14980923	1		
**Glo**	0.143774356	−0.066326113	1	
**MTN**	0.556604454	0.024529584	0.146238976	1

**Table 7:** ANOVA for CSSR.

	**Source of variation**	**Sum of squares**	**Degree of freedom**	**Mean squares**	**F statistic**	***P*-value**
**Airtel**	Quarters	3.080164	3	1.026721	5.623495	0.003264
	Error	5.842467	32	0.182577		
	Total	8.922631	35			
**Etisalat**	Quarters	0.059275	3	0.019758	0.584086	0.629807
	Error	1.082489	32	0.033828		
	Total	1.141764	35			
**Glo**	Quarters	0.421	3	0.140333	1.349765	0.275728
	Error	3.327	32	0.103969		
	Total	3.748	35			
**MTN**	Quarters	2.207208	3	0.735736	1.321611	0.284484
	Error	17.81429	32	0.556697		
	Total	20.0215	35

**Table 8:** ANOVA for DCR.

	**Source of variation**	**Sum of squares**	**Degree of freedom**	**Mean squares**	**F statistic**	***P*-value**
**Airtel**	Quarters	0.031631	3	0.010544	2.646908	0.065774
	Error	0.127467	32	0.003983		
	Total	0.159097	35			
**Etisalat**	Quarters	0.021978	3	0.007326	0.491032	0.690992
	Error	0.477422	32	0.014919		
	Total	0.4994	35			
**Glo**	Quarters	0.047808	3	0.015936	0.277619	0.841112
	Error	1.836889	32	0.057403		
	Total	1.884697	35			
**MTN**	Quarters	0.117533	3	0.039178	0.452483	0.717324
	Error	2.770689	32	0.086584		
	Total	2.888222	35

**Table 9:** ANOVA for SDCCH congestion.

	**Source of variation**	**Sum of squares**	**Degree of freedom**	**Mean squares**	**F statistic**	***P*-value**
**Airtel**	Quarters	0.1965	3	0.0655	2.468528	0.079868
	Error	0.849089	32	0.026534		
	Total	1.045589	35			
**Etisalat**	Quarters	0.016942	3	0.005647	1.856197	0.156919
	Error	0.097356	32	0.003042		
	Total	0.114297	35			
**Glo**	Quarters	0.523389	3	0.174463	0.289155	0.83288
	Error	19.30733	32	0.603354		
	Total	19.83072	35			
**MTN**	Quarters	0.103631	3	0.034544	1.299782	0.291458
	Error	0.850444	32	0.026576		
	Total	0.954075	35

**Table 10:** ANOVA for TCH congestion.

	**Source of variation**	**Sum of squares**	**Degree of freedom**	**Mean squares**	**F statistic**	***P*-value**
**Airtel**	Quarters	0.610178	3	0.203393	4.641974	0.008351
	Error	1.402111	32	0.043816		
	Total	2.012289	35			
**Etisalat**	Quarters	0.141878	3	0.047293	2.084203	0.121863
	Error	0.726111	32	0.022691		
	Total	0.867989	35			
**Glo**	Quarters	0.056511	3	0.018837	0.126161	0.943923
	Error	4.777889	32	0.149309		
	Total	4.8344	35			
**MTN**	Quarters	0.093267	3	0.031089	0.467	0.707347
	Error	2.130289	32	0.066572		
	Total	2.223556	35

**Table 11:** Tukey's multiple comparison post hoc test for CSSR.

**Mobile network operator**	**Quarter**	**Quarter**	**Mean difference**	**Lower limit (95% confidence intervals)**	**Upper limit (95% confidence intervals)**	***P*-value**
**Airtel**	1	2	−0.6249	−0.2911	0.0427	0.1029
	1	3	−0.9271	−0.5933	−0.2596	0.0003
	1	4	−1.0993	−0.7656	−0.4318	0.0000
	2	3	−0.6360	−0.3022	0.0316	0.0858
	2	4	−0.8082	−0.4744	−0.1407	0.0034
	3	4	−0.5060	−0.1722	0.1616	0.4976
**Etisalat**	1	2	−0.1237	0.0867	0.2970	0.6711
	1	3	−0.2259	−0.0156	0.1948	0.9969
	1	4	−0.1604	0.0500	0.2604	0.9125
	2	3	−0.3126	−0.1022	0.1081	0.5472
	2	4	−0.2470	−0.0367	0.1737	0.9626
	3	4	−0.1448	0.0656	0.2759	0.8253
**Glo**	1	2	−0.4507	−0.0689	0.3129	0.9588
	1	3	−0.6741	−0.2922	0.0896	0.1782
	1	4	−0.5141	−0.1322	0.2496	0.7756
	2	3	−0.6052	−0.2233	0.1585	0.3903
	2	4	−0.4452	−0.0633	0.3185	0.9675
	3	4	−0.2218	0.1600	0.5418	0.6594
**MTN**	1	2	−0.3759	−0.0756	0.2248	0.8984
	1	3	−0.5104	−0.2100	0.0904	0.2431
	1	4	−0.9404	−0.6400	−0.3396	0.0000
	2	3	−0.4348	−0.1344	0.1659	0.6116
	2	4	−0.8648	−0.5644	−0.2641	0.0001
	3	4	−0.7304	−0.4300	−0.1296	0.0031

**Table 12:** Tukey's multiple comparison post hoc test for DCR.

**Mobile network operator**	**Quarter**	**Quarter**	**Mean difference**	**Lower limit (95% confidence intervals)**	**Upper limit (95% confidence intervals)**	***P*-value**
**Airtel**	1	2	−0.0642	0.0000	0.0642	1.0000
	1	3	−0.1197	−0.0556	0.0086	0.1066
	1	4	−0.0386	0.0256	0.0897	0.6939
	2	3	−0.1197	−0.0556	0.0086	0.1066
	2	4	−0.0386	0.0256	0.0897	0.6939
	3	4	0.0169	0.0811	0.1453	0.0096
**Etisalat**	1	2	−0.1442	−0.0522	0.0397	0.4154
	1	3	−0.1386	−0.0467	0.0453	0.5113
	1	4	−0.1575	−0.0656	0.0264	0.2281
	2	3	−0.0864	0.0056	0.0975	0.9983
	2	4	−0.1053	−0.0133	0.0786	0.9778
	3	4	−0.1108	−0.0189	0.0730	0.9410
**Glo**	1	2	−0.1703	0.0156	0.2014	0.9955
	1	3	−0.0925	0.0933	0.2792	0.5203
	1	4	−0.1292	0.0567	0.2425	0.8344
	2	3	−0.1081	0.0778	0.2637	0.6604
	2	4	−0.1448	0.0411	0.2270	0.9279
	3	4	−0.2225	−0.0367	0.1492	0.9472
**MTN**	1	2	−0.1742	−0.0078	0.1587	0.9992
	1	3	−0.1498	0.0167	0.1831	0.9924
	1	4	−0.0331	0.1333	0.2998	0.1492
	2	3	−0.1420	0.0244	0.1909	0.9770
	2	4	−0.0253	0.1411	0.3076	0.1172
	3	4	−0.0498	0.1167	0.2831	0.2411

**Table 13:** Tukey's multiple comparison post hoc test for SDCCH congestion.

**Mobile network operator**	**Quarter**	**Quarter**	**Mean difference**	**Lower limit (95% confidence intervals)**	**Upper limit (95% confidence intervals)**	***P*-value**
**Airtel**	1	2	−0.0466	0.0567	0.1600	0.4454
	1	3	0.0667	0.1700	0.2733	0.0007
	1	4	0.0678	0.1711	0.2744	0.0007
	2	3	0.0100	0.1133	0.2166	0.0278
	2	4	0.0112	0.1144	0.2177	0.0260
	3	4	−0.1022	0.0011	0.1044	1.0000
**Etisalat**	1	2	−0.1068	−0.0489	0.0091	0.1198
	1	3	−0.0546	0.0033	0.0613	0.9985
	1	4	−0.0580	0.0000	0.0580	1.0000
	2	3	−0.0057	0.0522	0.1102	0.0879
	2	4	−0.0091	0.0489	0.1068	0.1198
	3	4	−0.0613	−0.0033	0.0546	0.9985
**Glo**	1	2	−0.7016	−0.2344	0.2327	0.5208
	1	3	−0.4483	0.0189	0.4860	0.9995
	1	4	−0.3827	0.0844	0.5516	0.9586
	2	3	−0.2138	0.2533	0.7205	0.4554
	2	4	−0.1483	0.3189	0.7860	0.2616
	3	4	−0.4016	0.0656	0.5327	0.9798
**MTN**	1	2	−0.2492	−0.0556	0.1381	0.8576
	1	3	−0.0992	0.0944	0.2881	0.5442
	1	4	−0.1847	0.0089	0.2025	0.9993
	2	3	−0.0436	0.1500	0.3436	0.1702
	2	4	−0.1292	0.0644	0.2581	0.7955
	3	4	−0.2792	−0.0856	0.1081	0.6213

**Table 14:** Tukey's multiple comparison post hoc test for TCH congestion.

**Mobile network operator**	**Quarter**	**Quarter**	**Mean difference**	**Lower limit (95% confidence intervals)**	**Upper limit (95% confidence intervals)**	***P*-value**
**Airtel**	1	2	0.0165	0.1533	0.2902	0.0241
	1	3	0.1587	0.2956	0.4324	0.0000
	1	4	0.1920	0.3289	0.4657	0.0000
	2	3	0.0054	0.1422	0.2791	0.0396
	2	4	0.0387	0.1756	0.3124	0.0085
	3	4	−0.1035	0.0333	0.1702	0.9067
**Etisalat**	1	2	−0.1790	0.0233	0.2257	0.9886
	1	3	−0.0679	0.1344	0.3368	0.2830
	1	4	−0.0645	0.1378	0.3401	0.2635
	2	3	−0.0912	0.1111	0.3134	0.4445
	2	4	−0.0879	0.1144	0.3168	0.4191
	3	4	−0.1990	0.0033	0.2057	1.0000
**Glo**	1	2	−0.2833	0.0256	0.3345	0.9957
	1	3	−0.2133	0.0956	0.4045	0.8284
	1	4	−0.2256	0.0833	0.3922	0.8782
	2	3	−0.2389	0.0700	0.3789	0.9230
	2	4	−0.2511	0.0578	0.3667	0.9545
	3	4	−0.3211	−0.0122	0.2967	0.9995
**MTN**	1	2	−0.2638	−0.0244	0.2149	0.9920
	1	3	−0.2226	0.0167	0.2560	0.9974
	1	4	−0.1293	0.1100	0.3493	0.5913
	2	3	−0.1982	0.0411	0.2804	0.9641
	2	4	−0.1049	0.1344	0.3738	0.4250
	3	4	−0.1460	0.0933	0.3326	0.7072

**Table 15:** CSSR data for months and quarters of year 2014–2016.

**Year**	**Month**	**Quarter**	**Airtel**	**Etisalat**	**Glo**	**MTN**
**2014**	Jan	1	96.99	99.2	96.89	96.85
	Feb	1	98.09	99.26	98.04	96.94
	Mar	1	98.29	98.97	98.33	97.19
	Apr	2	97.87	99.03	97.23	97.11
	May	2	98.04	98.39	98.28	97.01
	Jun	2	98.08	99.23	98.08	97.12
	Jul	3	98.08	99.33	98.21	97.12
	Aug	3	98.33	99.07	98.15	97.42
	Sep	3	98.27	99.28	98.42	97.52
	Oct	4	98.64	99.04	98.17	97.56
	Nov	4	98.71	99.1	98.25	98.73
	Dec	4	98.45	99.09	98.35	98.78
**2015**	Jan	1	96.8	98.94	98.26	98.25
	Feb	1	96.72	99.22	98.3	98.43
	Mar	1	97.23	99.19	98.56	98.28
	Apr	2	97.34	99.13	98.4	98.59
	May	2	97.41	99.18	98.47	98.14
	Jun	2	97.39	99.12	98.22	98.24
	Jul	3	98.06	99.27	98.22	98.37
	Aug	3	97.92	99.28	98.28	98.51
	Sep	3	98.03	99.26	98.14	98.45
	Oct	4	97.95	99.24	98.13	98.86
	Nov	4	98.13	99.3	98.08	98.83
	Dec	4	98.36	99.2	98.09	98.72
**2016**	Jan	1	98.23	99.34	98	98.88
	Feb	1	97.8	99.38	98.2	98.82
	Mar	1	98.35	99.33	97.99	98.98
	Apr	2	98.42	99.39	98.03	99.05
	May	2	98.14	99.22	98.09	99.02
	Jun	2	98.43	99.36	98.39	99.02
	Jul	3	98.53	99.27	98.59	99.06
	Aug	3	98.34	98.98	98.65	99.08
	Sep	3	98.28	99.23	98.54	98.98
	Oct	4	98.37	98.99	98.33	98.95
	Nov	4	98.42	99.24	98.25	98.99
	Dec	4	98.36	99.18	98.11	98.96

**Table 16:** DCR data for months and quarters of year 2014–2016.

**Year**	**Month**	**Quarter**	**Airtel**	**Etisalat**	**Glo**	**MTN**
**2014**	Jan	1	0.84	0.55	1.19	1.21
	Feb	1	0.71	0.54	0.85	1.29
	Mar	1	0.6	0.57	0.83	1.19
	Apr	2	0.67	0.54	1.43	1.43
	May	2	0.74	0.68	0.78	1.33
	Jun	2	0.8	0.55	0.85	1.3
	Jul	3	0.82	0.59	0.81	1.23
	Aug	3	0.85	0.6	0.78	1.22
	Sep	3	0.82	0.58	0.81	1.16
	Oct	4	0.79	0.86	0.91	1.23
	Nov	4	0.75	0.84	0.96	0.78
	Dec	4	0.73	0.8	0.86	0.72
**2015**	Jan	1	0.82	0.53	0.5	1.02
	Feb	1	0.84	0.51	0.46	0.9
	Mar	1	0.79	0.53	0.87	0.5
	Apr	2	0.69	0.52	0.48	0.85
	May	2	0.72	0.53	0.53	0.93
	Jun	2	0.75	0.54	0.4	0.72
	Jul	3	0.74	0.5	0.41	0.72
	Aug	3	0.71	0.5	0.55	0.76
	Sep	3	0.75	0.54	0.53	0.78
	Oct	4	0.7	0.54	0.46	0.72
	Nov	4	0.62	0.48	0.41	0.82
	Dec	4	0.63	0.44	0.6	0.82
**2016**	Jan	1	0.65	0.46	0.5	0.67
	Feb	1	0.69	0.43	0.5	0.71
	Mar	1	0.65	0.43	0.57	0.5
	Apr	2	0.69	0.72	0.54	0.45
	May	2	0.76	0.47	0.57	0.5
	Jun	2	0.77	0.47	0.55	0.55
	Jul	3	0.76	0.46	0.55	0.68
	Aug	3	0.86	0.7	0.49	0.64
	Sep	3	0.78	0.5	0.5	0.65
	Oct	4	0.73	0.27	0.54	0.63
	Nov	4	0.71	0.47	0.5	0.49
	Dec	4	0.7	0.44	0.52	0.58

**Table 17:** SDCCH congestion data for months and quarters of year 2014–2016.

**Year**	**Month**	**Quarter**	**Airtel**	**Etisalat**	**Glo**	**MTN**
**2014**	Jan	1	0.4	0.1	0.58	0.17
	Feb	1	0.14	0.07	0.24	0.17
	Mar	1	0.09	0.08	0.28	0.1
	Apr	2	0.2	0.23	1.51	0.68
	May	2	0.16	0.09	0.24	0.12
	Jun	2	0.12	0.09	0.22	0.12
	Jul	3	0.11	0.08	0.17	0.1
	Aug	3	0.16	0.13	0.23	0.09
	Sep	3	0.11	0.09	0.16	0.08
	Oct	4	0.09	0.07	0.17	0.13
	Nov	4	0.12	0.09	0.15	0.1
	Dec	4	0.12	0.11	0.16	0.12
						
**2015**	Jan	1	0.64	0.16	0.14	0.53
	Feb	1	0.79	0.11	0.13	0.48
	Mar	1	0.49	0.14	0.41	0.16
	Apr	2	0.5	0.29	0.14	0.39
	May	2	0.6	0.33	1.38	0.73
	Jun	2	0.51	0.13	1.55	0.13
	Jul	3	0.26	0.12	1.7	0.12
	Aug	3	0.34	0.11	1.49	0.14
	Sep	3	0.14	0.13	1.52	0.14
	Oct	4	0.36	0.13	1.78	0.11
	Nov	4	0.19	0.12	1.79	0.34
	Dec	4	0.2	0.14	1.86	0.42
**2016**	Jan	1	0.19	0.12	2.19	0.15
	Feb	1	0.2	0.11	1.94	0.15
	Mar	1	0.21	0.09	2.32	0.11
	Apr	2	0.17	0.03	1.86	0.1
	May	2	0.18	0.13	1.75	0.12
	Jun	2	0.2	0.1	1.69	0.13
	Jul	3	0.16	0.12	1.54	0.17
	Aug	3	0.18	0.07	0.71	0.14
	Sep	3	0.16	0.1	0.54	0.19
	Oct	4	0.18	0.13	0.5	0.27
	Nov	4	0.17	0.09	0.48	0.25
	Dec	4	0.18	0.1	0.58	0.2

**Table 18:** TCH congestion data for months and quarters of year 2014–2016.

**Year**	**Month**	**Quarter**	**Airtel**	**Etisalat**	**Glo**	**MTN**
**2014**	Jan	1	0.79	0.27	0.79	0.55
	Feb	1	0.32	0.29	0.69	0.57
	Mar	1	0.32	0.55	1.05	0.42
	Apr	2	0.45	0.18	1.67	1.27
	May	2	0.29	0.98	0.58	0.43
	Jun	2	0.28	0.09	0.99	0.37
	Jul	3	0.32	0.12	0.94	0.31
	Aug	3	0.23	0.24	1.06	0.3
	Sep	3	0.25	0.14	0.69	0.29
	Oct	4	0.12	0.08	0.72	0.34
	Nov	4	0.15	0.09	0.64	0.36
	Dec	4	0.14	0.12	0.73	0.31
**2015**	Jan	1	0.91	0.39	1.29	0.6
	Feb	1	0.89	0.19	1.06	0.64
	Mar	1	0.99	0.19	0.6	1.09
	Apr	2	0.99	0.26	0.93	0.56
	May	2	0.64	0.27	0.7	0.77
	Jun	2	0.73	0.26	1.25	0.75
	Jul	3	0.43	0.18	1.44	0.63
	Aug	3	0.47	0.2	1.5	0.88
	Sep	3	0.52	0.19	1.58	1.06
	Oct	4	0.46	0.17	1.53	0.49
	Nov	4	0.41	0.19	1.52	0.4
	Dec	4	0.4	0.26	1.69	0.4
**2016**	Jan	1	0.48	0.33	1.74	0.28
	Feb	1	0.48	0.28	1.46	0.3
	Mar	1	0.39	0.24	1.56	0.27
	Apr	2	0.27	0.1	1.54	0.25
	May	2	0.3	0.19	1.28	0.25
	Jun	2	0.24	0.19	1.07	0.29
	Jul	3	0.19	0.15	0.78	0.39
	Aug	3	0.19	0.15	0.63	0.34
	Sep	3	0.31	0.15	0.76	0.37
	Oct	4	0.29	0.17	0.83	0.38
	Nov	4	0.31	0.18	0.86	0.5
	Dec	4	0.33	0.23	0.97	0.55

## References

[1] NCC, A Report on the Network Quality of Service and Performance of the GSM Networks in Nigeria, in: Guardian Newspaper of Nigeria (Ed.), 2005.

[2] NCC (2014). Year End Subscriber/Network Data Report for Telecommunications Operating Companies in Nigeria.

[3] O. Oyetunji, Improving call setup success rate in GSM service area using RF optimisation, in: Proceedings of the 11th International Conference on Electronics, Computer and Computation (ICECCO 2014), 2014, pp. 1–4.

[4] Ozovehe A., Usman A. (2015). Performance analysis of GSM networks in Minna Metropolis of Nigeria. Niger. J. Technol..

[5] Lawal B., Ukhurebor K., Adekoya M., Aigbe E. (2016). Quality of service and performance analysis of a GSM Network in Eagle Square, Abuja and its Environs, Nigeria. Int. J. Sci. Eng. Res..

[6] M.A. Salman, S.I. Popoola, N. Faruk, N.T. Surajudeen-Bakinde, A.A. Oloyede, L.A. Olawoyin, Adaptive Neuro-Fuzzy model for path loss prediction in the VHF band, in: Proceedings of the 2017 International Conference on Computing Networking and Informatics (ICCNI), Lagos, Nigeria, 2017, pp. 1–6. 〈http://dx.doi.org/10.1109/ICCNI.2017.8123768〉.

[7] A. Ozovehe, O.U. Okereke, E. Anene, A.U. Usman, Traffic congestion analysis in mobile macrocells, in: Proceedings of the International Conference on Information and Communication Technology and Its Applications (ICTA 2016) Federal University of Technology, Minna, Nigeria, 28–30 November 2016, pp. 243–249.

[8] Oseni O.F., Popoola S.I., Enumah H., Gordian A. (2014). Radio frequency optimization of mobile networks in Abeokuta, Nigeria for improved quality of service. Int. J. Res. Eng. Technol..

[9] I.A. Sikiru, N. Faruk, S.I. Popoola, Y. Imam-Fulani, A.A. Oloyede, L.A. Olawoyin, N.T. Surajudeen-Bakinde, Effects of detection threshold and frame size on duty cycle in GSM bands, in: Proceedings of the 3rd International Conference on Electro-Technology for National Development, Federal University of Technology (FUTO), Owerri, Imo State, Nigeria, 7–10 November 2017, pp. 343–346.

[10] Basha S.I., Shaik I. (2013). Reducing handover failure rate by RF optimization. Int. J. Eng. Innov. Technol..

[11] Laiho J., Wacker A., Novosad T. (2006). Radio Network Planning and Optimisation for UMTS.

[12] Mojisola D.F., Gbolahan K. (2015). Participatory analysis of cellular network quality of service. Int. J. Comput. ICT Res..

[13] Gerpott T.J., Rams W., Schindler A. (2001). Customer retention, loyalty, and satisfaction in the German mobile cellular telecommunications market. Telecommun. Policy.

[14] Khan M.A. (2010). An empirical assessment of service quality of cellular mobile telephone operators in Pakistan. Asian Soc. Sci..

[15] Paulrajan R., Rajkumar H. (2011). Service quality and customers preference of cellular mobile service providers. J. Technol. Manag. Innov..

[16] S.I. Popoola, J.A. Badejo, S.O. Ojewande, A.A. Atayero, Statistical evaluation of quality of service offered by GSM network operators in Nigeria, in: Lecture Notes in Engineering and Computer Science: Proceedings of the World Congress on Engineering 2017, San Francisco, USA, 25–27 October 2017, pp. 69–73.

[17] S.I. Popoola, A.A. Atayero, N. Faruk, C.T. Calafate, L.A. Olawoyin, V.O. Matthews, Standard propagation model tuning for path loss predictions in built-up environments, in: Proceedings of the International Conference on Computational Science and its Applications, 2017, pp. 363–375.

[18] S.I. Popoola, A.A. Atayero, N. Faruk, C.T. Calafate, E. Adetiba, V.O. Matthews, Calibrating the standard path loss model for urban environments using field measurements and geospatial data, in: Lecture Notes in Engineering and Computer Science: Proceedings of the World Congress on Engineering 2017, London, U.K., 5–7 July 2017, pp. 513–518.

